# Effect of facemasks on empathy and relational continuity: a randomised controlled trial in primary care

**DOI:** 10.1186/1471-2296-14-200

**Published:** 2013-12-24

**Authors:** Carmen Ka Man Wong, Benjamin Hon Kei Yip, Stewart Mercer, Sian Griffiths, Kenny Kung, Martin Chi-sang Wong, Josette Chor, Samuel Yeung-shan Wong

**Affiliations:** 1 JC School of Public Health and Primary Care, The Chinese University of Hong Kong, 4/F, School of Public Health building, Prince of Wales Hospital, Shatin, New Territories, Hong Kong SAR; 2 General Practice and Primary Care, Institute of Health and Wellbeing, College of Medical, Veterinary and Life Sciences, University of Glasgow, 1 Horseletthill Road, Glasgow G12 9LX, UK

**Keywords:** Public health, Primary health care, General practice, Continuity of patient care, Empathy

## Abstract

**Background:**

There is limited evidence to support the use of facemasks in preventing infection for primary care professionals. Negative effects on communication has been suggested when the physician wears a facemask. As communication skills and doctor patient relationship are essential to primary care consultations, the effects of doctor’s facemask wearing were explored.

**Method:**

A randomised controlled study was conducted in primary care to explore the effects of doctors wearing facemasks on patients’ perception of doctors’ empathy, patient enablement and patient satisfaction. Primary care doctors were randomized to mask wearing and non mask wearing clinical consultations in public primary care clinics in Hong Kong. Patients’ views were gathered using the Consultation and Relational Empathy (CARE) Measure, Patient Enablement Instrument (PEI) and an overall satisfaction rating scale. The effects of face mask wearing were investigated using multilevel (hierarchical) modelling.

**Results:**

1,030 patients were randomised to doctor-mask wearing consultations (n = 514) and non mask wearing consultations (n = 516). A significant and negative effect was found in the patients’ perception of the doctors’ empathy (CARE score reduction -0.98, p-value = 0.04). In the more established doctor-patient relationship, the effect of doctors’ mask wearing was more pronounced (CARE score reduction -5.67, p-value = 0.03).

**Conclusion:**

This study demonstrates that when doctors wearing a facemask during consultations, this has a significant negative impact on the patient’s perceived empathy and diminish the positive effects of relational continuity. Consideration should be taken in planning appropriate use of facemasks in infectious disease policy for primary care and other healthcare professionals at a national, local or practice level.

**Clinical trial registration:**

This trial was registered on Chinese Clinical Trial Register (ChiCTR). Registration no.: ChiCTR-TTRCC-12002519. URL: http://www.chictr.org/en/proj/show.aspx?proj=3486. Due to administrative error, registration of trial did not take place until after the trial started on 1^st^ August 2011 and registration number was released on 21^st^ September 2012.

## Background

Since the global outbreak of pandemic influenza and severe acute respiratory syndrome (SARS), wearing facemasks is common practice for healthcare providers in clinical settings in many countries in Asia. A facemask is a loose fitting disposable device covering the wearer’s nose and mouth and acts as a physical barrier to potential contaminants in the immediate environment but can have limited effectiveness in blocking small particles. There is little proven benefit in the sole use of facemask (without hand washing) to prevent healthcare personnel from contracting infections in randomized control trials [1-3], with research conducted during the SARS outbreak suggesting a potential negative impact of infection precaution measures including mask wearing, on the domain of doctors’ empathy of a patient satisfaction questionnaire [4].

The doctor-patient interaction is essential for optimum information exchange and medical decision making [5-7], and a crucial component of the quality of primary care consultations [8-10]. It has been shown that nonverbal communication is important for the therapeutic relationship [11] and is related to patients’ adherence to medical advice and medication compliance, patient satisfaction and positive clinical outcomes [12-16]. The importance of emotional perception, expression and reciprocity in non verbal communication was postulated to impact the outcome of the doctor-patient interaction [11]. Studies in non clinical contexts have shown that subliminal facial expressions can influence the viewer’s emotional state, attitudes and subsequent behaviours [17,18]. However, only a few studies have looked at the influence of facial expressions on patient health outcomes [14,19]; eye contact was a strong predictor of a positively rated doctor patient interaction [19], whilst doctors’ distancing behaviour, such as not smiling and looking away was perceived negatively by patients [14]. During hospital admissions, facial expressiveness (such as smiling, nodding, frowning) of physical therapists were associated with an improvement in ability to perform activities of daily living and also a decrease in confusion for elderly patients [14].

Within the doctor patient consultation, patient’s perception of doctor’s empathy has been shown to be essential in developing trust, communication and a therapeutic alliance [10,20,21]. Studies have demonstrated the impact of doctors’ empathy and patient centred care on patient enablement [22] and health outcomes [6,23] in both chronic and acute conditions [24-27]. In this study, a randomized control trial was conducted to explore the effects of facemask wearing among primary care doctors in Hong Kong on patients’ perception of the doctors’ empathy. We hypothesized that patients who consulted mask wearing doctors would report lower scores in doctors’ empathy when compared to non mask wearing doctors, due to physical obstruction of facial expressions and the subsequent impact on the perceived empathic response. We also explored the effects of facemask wearing on patient enablement and patient satisfaction. In addition, we hypothesized that the effect of facemask wearing is minimal when the patient knows the doctor well and there is an established therapeutic rapport.

## Methods

### Setting and study design

**Figure 1:**
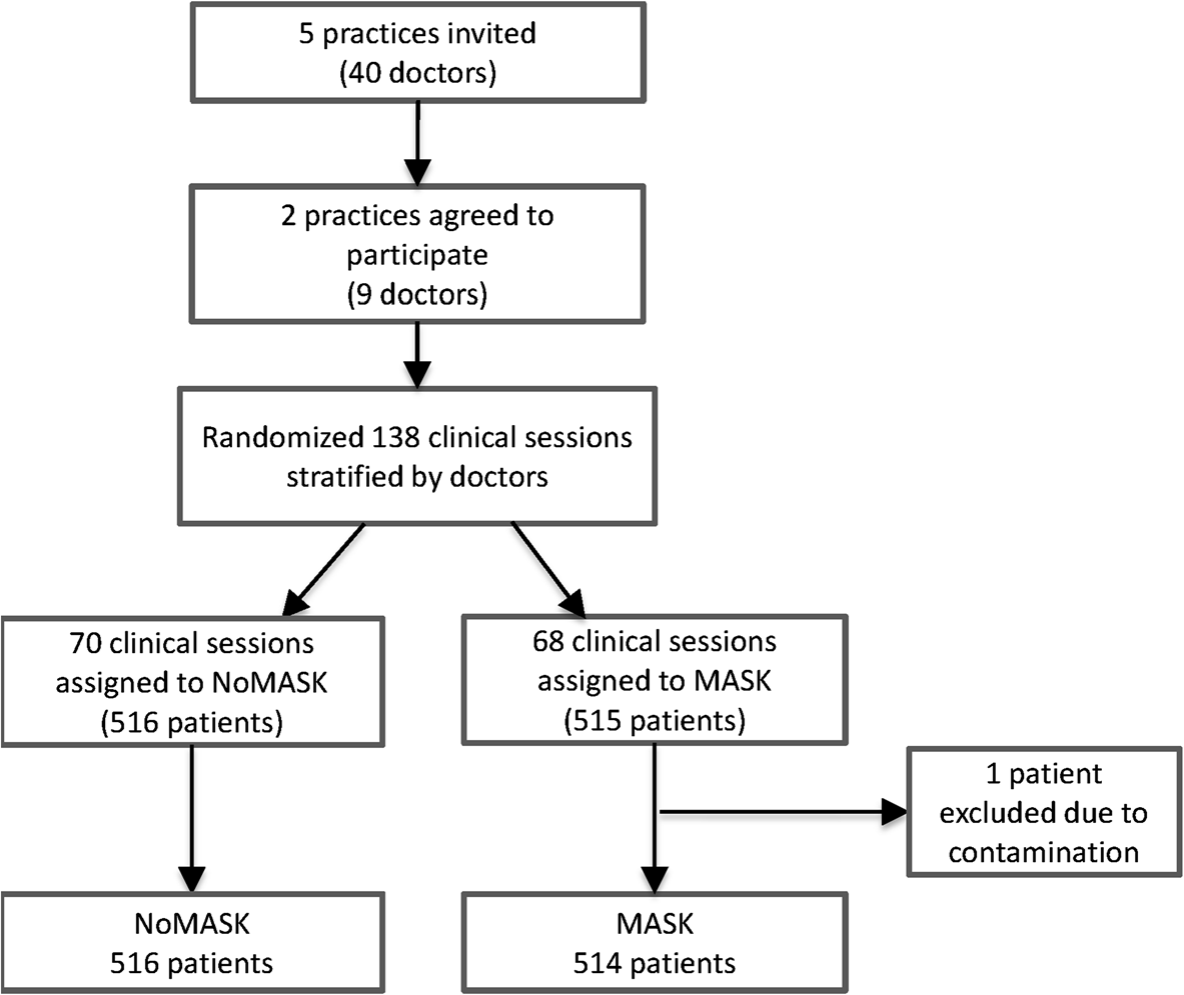
**Flowchart showing the recruitment and randomization of clinical sessions and mask-wearing.** MASK = mask-wearing, NoMASK = non mask wearing.

Five primary care clinics of the public sector (Hospital Authority) in the Shatin district, Hong Kong were invited to participate in this study of which two of the five clinics agreed to participate (Figure 1). The chosen study period was from August - September 2011 as to avoid the Hong Kong influenza season in which alerts are more likely to occur requiring doctors to wear masks in consultations.

All doctors (n = 40) were invited of whom nine doctors participated in the study. For each doctor, all sessions in the forthcoming monthly schedule were randomly assigned to mask wearing (MASK) or non mask wearing (NoMASK) sessions by a research assistant using computer software. At the commencement of clinical sessions on a particular morning or afternoon, the doctor was instructed to wear a facemask or not to wear a facemask and was required to follow that instruction throughout his/her clinical session. During each clinical session, a trained research assistant approached consecutive patients for their willingness to participate in the study and to obtain informed consent. Following the consultation, patients were required to complete a questionnaire about the consultation before leaving.

### Outcome measures

The primary outcome measure was the Consultation and Relational Empathy (CARE) Measure. The CARE measure is a patient-rated experience measure developed by Mercer et al. [27-29] which has undergone extensive validation. The Chinese version of the CARE measure has been found to be a reliable and valid tool to assess patient-rated empathy of doctors during consultation [30,31] and have been shown to be able to effectively differentiate between doctors in terms of patient-rated empathy [30]. Patients rated the ten questions in the CARE measures with score of 1 for 'poor’ and 5 for 'excellent’ to the questions, such as 'How was the doctor at making you feel at ease (item 1)’. The total CARE is calculated as an average mean item score multiplied by 10 (and can range from 10 to 50) [31]. Secondary outcome measures were: 1) patient satisfaction, in which patients rated their overall satisfaction with the doctor-patient interaction on a 7-point likert scale (1 = worst, 7 = excellent); 2) patient enablement, by use of the six-item patient enablement instrument (PEI) which measured the impact of patient doctor encounter on the patient’s ability to cope with and understand his/her illness [32] of which the Chinese version has been shown to have good validity and reliability [33]. Rating is on a 3-point scale (same or less/better/much better) and scored as 0, 1, 2. Total PEI score is calculated by the average of the scores of the applicable items multiplied by six [31]. Cases that had more than three 'non applicable’ items were excluded [33]. Thus total PEI score ranges from the lowest 0 score to the highest 12 score.

Factors shown to be related to CARE measure scores [30,31] were also included in the questionnaire which included the reason for consultation, self-assessed general health over the previous 12 months, familiarity of the doctor and patient’s self-reported consultations time length.

Information on the participating doctors was also collected and included doctor’s gender, age and training grade.

### Sample size

As previous studies have shown that at least 50 consultations were needed per doctor for the CARE Measure to differentiate differences in CARE Measure score between individual doctors [29], 100 consultations per doctor with a total of 1000 consultations were needed to detect significant differences in effect sizes between the CARE Measure score of mask-wearing doctors and non mask-wearing doctors. Given this sample size and with an alpha level equal 0.05 this study has 0.81 power to detect Cohen-d equal or more than 0.2 (i.e., small effect size), after adjusting the design effect by assuming the intraclass correlation is equal 0.1.

### Statistical analysis

To test the effect of wearing a facemask during clinical consultation on the outcome measures, we used linear mixed model for analysis. This can adjust for possible cluster effects of patients nested with doctors, as well as potential confounding effects from patients’ demographic variables, such as age and gender. Important independent predictors of CARE measure in the recent Hong Kong study of primary care patients [30] were pre-identified and included into the model. These four variables included patient’s general health in the past 12 months, familiarity with the doctor, the patient’s self reported consultation time and whether consulting for a new or old problem. Improvements in nested linear models were assessed using likelihood ratio test. For non-nested linear models, Akaike Information Criterion (AIC) was used. The restricted maximum likelihood was used to compare mixed models, given the same mean value structure. All linear mixed models were estimated with “nlme” and “lme4”, R-packages from the statistical software R [34].

## Results and discussion

During the study period, 1031 patients were recruited consecutively in the study sessions, of which only one patient was excluded from the analysis as the doctor was required to remove the facemask as the patient was unable to hear (Figure 1). All patients consulted the participating doctor only once during the study period. The number of patients participating per doctor ranged from 103 to 128 for the nine doctors. The characteristics of the patients are shown in Table 1. The gender and age group distribution of the participating patients were similar to previous Chinese-CARE measure studies in Hong Kong [30,31]. Between the MASK and NoMASK groups, age and education differed significantly. Thus age and education were included in the process of mixed model building to adjust for the potential confounding effect. The distributions of the four known important Chinese-CARE score predictors (general health, type of problem, knowing the doctor and consultation time length) were similar between both MASK and NoMASK groups, but were also included for the analysis as independent predictors.

**Table 1:** Characteristics of patients recruited in the doctor-mask wearing (MASK) and non mask wearing (NoMASK) clinical consultations

	**NoMASK**	**MASK**
	**No (%)**	**No (%)**
**Age**		
<44	77 (15.0)	60 (11.7)
45-64	251 (48.7)	215 (41.7)
>65	187 (36.3)	238 (46.2)
**Gender**		
Female	306 (59.4)	304 (59.0)
Male	209 (40.6)	207 (40.2)
**Education**		
Primary or Below	210 (40.9)	259 (50.9)
Above Primary	303 (59.1)	250 (49.1)
**General Health over last 12 months**		
Very bad/Bad	89 (17.4)	94 (18.4)
Fair	295 (57.7)	291 (56.9)
Good/Very Good	127 (24.9)	127 (24.9)
**Knowing the doctor**		
Not Well/Neutral	494 (96.0)	490 (95.1)
Quite Well/Very Well	21 (4.1)	23 (4.5)
**Nature of the problem**		
New (acute) illness	102 (19.8)	77 (15.0)
Old (chronic) illness	386 (74.8)	418 (81.5)
Both new and old	28 (5.4)	18 (3.5)
**Duration of consultation, mean (SD)**	7.64 (4.55)	7.67 (4.85)

The mean CARE score of the MASK group (33.93) was significantly lower (p = 0.04) than the mean of NoMASK group (34.91) (Table 2). There were no significant differences between mean scores of patient satisfaction and patient enablement between the two groups, which was confirmed by regression analysis. Table 2 illustrates the unadjusted mean score value of CARE, PEI and satisfaction of the two groups.

**Table 2:** Table showing CARE scores, patient enablement index (PEI) and patient satisfaction scores in doctor- mask wearing (MASK) and non mask wearing (NoMASK) clinical consultations

	**Mean ± standard deviation**		
	**NoMASK**	**MASK**	**Pr(>|t|)**
**Total CARE scores**	34.91 ± 7.84	33.93 ± 7.65	0.043
**PEI**	2.60 ± 2.53	2.56 ± 2.41	0.869
**Patient Satisfaction**	5.69 ± 0.95	5.62 ± 1.04	0.251

**Figure 2:**
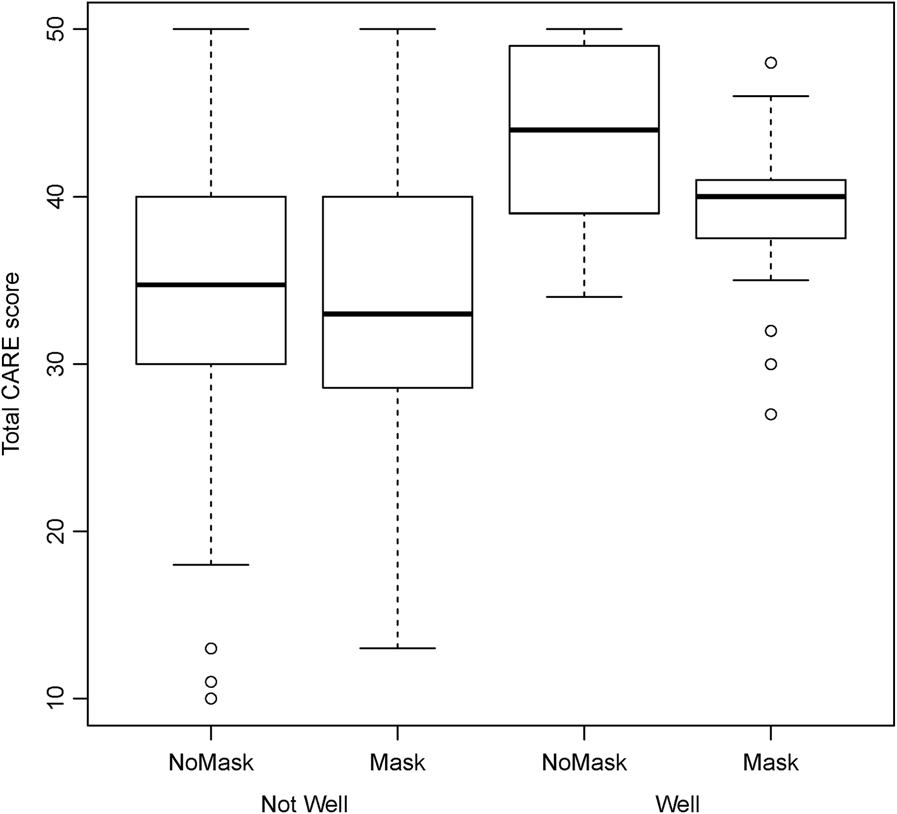
**Boxplot showing the effect of mask wearing and knowing the doctor on CARE score.** MASK = mask-wearing, NoMASK = non mask wearing.

Among the four established CARE related predictors, “knowing the doctor” was pre- identified as a doctor-related factor that may potentially interact with facemask wearing on CARE measure. Figure 2 shows the total raw CARE score plotted by mask status and “knowing the doctor”. Patients that were familiar with their doctor had, on average, higher CARE score than patients that were not familiar with their doctor (7.25 CARE score difference). Wearing a mask had little effect when the patient didn’t know the doctor well (-0.84 CARE score difference), but among patients who knew their doctor well, CARE scores were further reduced by (-4.71 CARE score difference). This suggests a potential interaction effect.

### Model building

Regression analysis was conducted where CARE score was the dependent variable. Age-group (linear with <45 coded as 0, and >65 as 2), education level (primary or below = 0, above primary = 1), and the cluster variables (clinic, doctor’s gender, doctor) were included in the model building procedure. Prior known important predictors, general health in the past 12 months (linear with good = 0, normal = 1, bad = 2), “knowing the doctor” (not knowing the doctor well/neutral = 0, quite well/very well = 1), self-reported consultation time length (mean centred), and the nature of the problem ('New Problem’, 'Old Problem’ and 'Both new and old problem’) were modelled as independent variables. The final model was a linear mixed model, where doctors were treated as random variable using random slope modelling, and excluded education level and doctor’s gender (Table 3). After adjustment of other variables the negative effect of wearing a facemask (-0.95) remained significant (see model A). Poor general health in the past 12 months was linearly associated with a lower CARE score (-1.51). Knowing the doctor quite well or very well had the highest positive impact (4.36) on CARE score. In addition, per minute of self reported consultation time, CARE score also increased by 0.32.

**Table 3:** Table showing mixed regression modelling of factors associated with CARE Measure score (model A) and with interaction effect (IE) of mask wearing on patients’ familiarity with the doctor (model B)

	**Best fitted model A without interaction**			**Best fitted model B with interaction**		
**Variable**	**Estimate**	**SE**	P-value*	**Estimate**	**SE**	**P-value**
Intercept^#^	35.76	1.07		35.64	1.06	
MASK	-0.95	0.44	0.027	-0.76	0.45	0.0836
Age	0.42	0.33	0.201	0.42	0.33	0.1948
General Health	-1.51	0.34	<0.0001	-1.51	0.34	<0.0001
Knowing the doctor well	4.36	1.14	<0.0001	6.94	1.63	<0.0001
Consultation time	0.32	0.05	<0.0001	0.32	0.05	<0.0001
Disease:Old	-0.34	0.60	0.5704	-0.36	0.60	0.5948
Disease:Both	1.92	1.19	0.1130	1.82	1.18	0.1342
MASK-Knowing the doctor well				-4.91	2.22	0.0308
Variance	Between^		5.86 (2.42)			5.81 (2.41)
component (SD)	Within		47.00 (6.86)			46.82 (6.84)

^#^Reference group is patient aged <45 years old, the consulting doctor didn’t wear mask, good self-reported health, didn’t know the doctor well, average consultation time is 7.66 minutes, the consultation was for new (acute) illness.

*P-values were obtained by permutation test on 10,000 permuted sample.

^The variance component of doctor random effect.

### Interaction analysis: MASK – “Knowing the doctor”

Model B (in Table 3) shows the interactive effects of mask wearing and “knowing the doctor well”. The coefficient estimation of other variables remained approximately the same after the adjustment of other variables (age, general health in the past 12 months, “knowing the doctor”, self-reported consultation time length and the nature of the problem), which indicated that the interaction was independent of the above variables. The effect of facemask wearing among patients who did not know his/her doctor well was minor and did not reach significance (CARE score reduction -0.75, p-value = 0.08). However, the positive effect of “knowing the doctor” (+6.94) on CARE score estimate was greatly diminished when the doctor wore a facemask with a reduction in CARE score estimate of 5.67 (Total mask effect on CARE score = -0.76-4.91 = -5.67, p-value = 0.03). Thus the CARE score estimate of 42.58 when the patient knows the doctor well is reduced to 36.91 (13.3% drop) when the doctor wore a facemask.

The random effect of doctors was assumed to be normally distributed with mean 0. The variances are estimated and presented in Table 3. The variability of CARE score could only partly be explained by clustering effect of doctors: estimated variance component of the between doctor random effect were 5.86 with an intraclass-correlation of 0.11. The model with the added interaction has approximate same estimates (variance component = 5.81, intraclass-correlation = 0.11).

### Comparison with existing literature

The mean CARE score of the NoMASK group (34.91) appears consistent with a previous study in Hong Kong [30] showing a mean of 34.6. Findings relating to CARE scores were also consistent; patients were more likely to rate empathy higher if they were familiar with the doctor or reported a longer consultation time and patients who rated poorer self reported health were more likely to give lower CARE scores [30,31].

Overall, patients rated high satisfaction in both groups (mean 5.6 on maximum scale of 7) and no significant association was found between facemask wearing and patient satisfaction. As patient expectations contributes greatly to the rating of patient satisfaction, facemask wearing appears to have neither a positive nor negative effect on patient satisfaction, which may reflect, in part cultural tolerance to mask wearing following the SARS epidemic in 2003 and the widespread use of facemasks in health care settings. Patient enablement was poor in both groups (PEI mean score 2.6, maximum 12) and similar to primary care patients in the UK (PEI mean score 3) [22].

Contrary to our hypothesis that established relational continuity and “knowing the doctor well” would be protective measures to the negative effects of mask wearing, it was in these groups of patients that the effects were more pronounced. “Knowing the doctor well” does have a marked positive effect on the CARE score, but this effect is almost mitigated when the doctors wore a facemask. The 5.67 drop in CARE score measure is likely to be clinically significant, as a 5 point drop in CARE score in the original study in the UK was able to differentiate between significantly below average doctors and significantly above average doctors [29].

### Strengths and limitations

This was the first study to explore wearing facemasks within the primary care consultation and its effect on empathy. Amongst the wealth of literature analyzing non-verbal behaviour and its effect on the doctor-patient relationship, this is the first in exploring the impact of concealing facial expressions on the patient’s perception of empathy. This study appears to strengthen the theory that emotional perception, expression and reciprocity is important in non-verbal communication which can affect the outcome of the doctor-patient interaction which was postulated by Roter et al. [11].

The findings of the study may be strengthened by increasing the number of clinics and extending the study into private practice settings. Given the significance of the interaction effect of doctor familiarity and facemask wearing in subgroup analysis, further studies in other health care systems where the doctor-patient interactions are more stable can explore whether this phenomenon is consistent or an effect of habit disruption of seeing his/her doctor with or without a mask.

In addition, randomization was executed on a sessional basis. For practical reasons, doctors cannot practically be blinded to the MASK or NoMASK allocation but randomisation and concealed allocation on an individual patient basis could reduce any potential variation in doctor’s behaviour (e.g., fatigue) and also reduce interviewer bias. However, this would be more disruptive to the clinic session and may be more problematic in raising doctor’s awareness and may intrinsically induce performance bias.

## Conclusion

### Summary

In this large randomized controlled trial, we found that the wearing of facemasks by doctors had little effect on patient enablement and satisfaction but had a significant and negative effect on patients’ perceptions of the doctors’ empathy. (33.93-34.91 = -0.98, p-value = 0.04). In subgroup analysis of whom patients reported an established doctor-patient relationship, the effect of doctors’ mask wearing was more pronounced (CARE score reduction -5.67, p-value = 0.03).

### Implications for research and practice

#### Communicating with patients

Identifying specific non verbal behaviours that enhance relational empathy and continuity could yield important tools for an effective therapeutic relationship in optimising a patient’s health outcomes. Further studies into the complexities of the doctor patient relationship could explore doctors’ own experience of facemask use on consulting behaviour and patient care along with other health professionals that have a continuous therapeutic relationship with patients (e.g., nurses, counsellors etc.). In addition, further studies to look into the role of facial expressions and micro-expressions, and the effect of concealment of expressions in emotional exchange in communication, may be particularly relevant in some cultures requiring veiling of the face (e.g., burka in muslim women) or clinical situations where empathy is essential (e.g., palliative care).

#### Infection control measures

The findings of this study are important in weighing up the benefits and risks of protective facemasks within doctor patient consultations and daily clinical practice. Facemasks offer limited protection in preventing infection [3] and aerosol transmission [35] through mucous membranes (i.e., conjunctivae). Meanwhile, a negative impact on the patient’s perceived empathy and relational continuity can reduce potential therapeutic effects such as decreased depression, improved immune response, improved quality of life and improved health outcomes [36,37]. In some countries and clinical institutions where facemask wearing has become mandatory and universal, review of current policies may be warranted in light of our current findings. For countries in which wearing facemasks is uncommon, care must be taken in conveying effective infection risk advice to healthcare professionals and caution in adopting guidelines regarding universal mask use (e.g., flu epidemics) particularly for medical physicians or other healthcare professionals where optimization of the therapeutic relationship is essential.

### Ethics approval

Ethical approval was obtained from the Joint CUHK-NTEC Clinical Research Ethical Committee (Ref. no.: CRE-2011.306-T) before the start of the trial.

## Competing interests

The authors declare that they have no competing interest.

## Authors’ contributions

All authors participated in the design of the study. SW-M, SYS-W, KK designed data collection tools and monitored data collection for the whole trial. CKM-W and BHK-Y drafted the manuscripts and performed data analysis. CKM-W, SW-M and SYS-W revised the draft paper. All authors interpreted the results and approved the final manuscript.

## Pre-publication history

The pre-publication history for this paper can be accessed here:

http://www.biomedcentral.com/1471-2296/14/200/prepub
